# Knowledge translation initiatives at the Transitional Pain Service: insights from healthcare provider outreach and patient education

**DOI:** 10.1186/s12913-025-12301-y

**Published:** 2025-01-29

**Authors:** Anna M. Lomanowska, Rabia Tahir, Christina Choo, Sabrina Zhu, Dora Y. Wang, P. Maxwell Slepian, Joel Katz, Hance Clarke

**Affiliations:** 1 Pain Research Unit, Department of Anesthesia and Pain Management, Toronto General Hospital, University Health Network, Toronto, ON Canada; 2 Transitional Pain Service, Toronto General Hospital, University Health Network, Toronto, ON Canada; 3 Michael G. DeGroote School of Medicine, McMaster University, Hamilton, ON Canada; 4 Department of Anesthesiology and Pain Medicine, University of Toronto, Toronto, ON Canada; 5 Department of Psychology, York University, Toronto, ON Canada

**Keywords:** Knowledge translation, Chronic post-surgical pain, Patient education, Healthcare provider education

## Abstract

Evidence-based treatment of chronic pain requires a multidisciplinary approach grounded in the biopsychosocial model. Implementing this approach within health systems relies on its acceptance by both healthcare providers and patients. While pioneering multidisciplinary pain clinics can serve as a model for implementation, a systematic effort is needed to share knowledge effectively and broadly. In the current paper we provide an overview of the knowledge translation initiatives undertaken at our Transitional Pain Service (TPS) at Toronto General Hospital, a state-of-the-art multidisciplinary pain program established in 2014 for patients at risk of developing chronic pain after surgery. The TPS team strives to enhance acceptance of this model of care among patients and providers, facilitate the establishment of similar clinics, and promote patient understanding of the integrated multidisciplinary pain care approach. Guided by the Knowledge to Action (KTA) framework, knowledge translation activities undertaken by our TPS team include clinician training, resources and outreach activities for providers, and patient education. Resource development was preceded by consultation and needs assessment among patients and providers and feedback from both groups was incorporated as part of the development process. The tailored resources were disseminated via the TPS clinic website and monitoring of online usage enables continuous evaluation of engagement. Barriers to engagement with the resources were examined through patient surveys and staff interviews. Based on these activities, we offer insights gained by our team throughout the knowledge translation process and provide recommendations for other clinical teams who wish to undertake similar initiatives.

**Supplementary Information:**

The online version contains supplementary material available at 10.1186/s12913-025-12301-y.

## Introduction

The medical treatment of chronic pain is undergoing a shift towards an evidence-based multidisciplinary approach rooted in the biopsychosocial model, which incorporates medical management alongside physical rehabilitation and psychological support [1, 2]. Implementing this approach, however, can pose challenges for pain clinics. They require resources to support a multidisciplinary team and to educate both healthcare providers and patients about effective pain management strategies that go beyond medical procedures and pain medication [1, 3]. Pioneering pain clinics that have successfully adopted this multidisciplinary approach serve as a model for other health services to follow and can provide valuable resources for other healthcare providers to learn from [4, 5]. However, a concerted knowledge translation initiative is required to effectively share knowledge. The aim of the current paper is to describe the knowledge translation activities undertaken by the Transitional Pain Service (TPS) at Toronto General Hospital, a state-of-the art multidisciplinary pain program for patients at risk of developing chronic pain and pain-related disability following a surgical procedure. We first provide a brief overview of the transitional pain care model adopted at our clinic and its impact so far. We then describe our knowledge translation initiatives geared towards healthcare providers and patients to increase awareness and acceptance of the multidisciplinary care model. Finally, we discuss our main insights and recommendations based on this work. We hope that the lessons learned from these initiatives will provide valuable insights for other teams implementing novel models of pain care.

## The multidisciplinary model of transitional pain care

### The need for a Transitional Pain Service

Pain is commonly experienced after having surgery and usually resolves within several weeks of a surgical procedure [6]. When managed appropriately, post-surgical pain does not have a lasting negative impact on a patient’s functioning or quality of life [6, 7]. However, approximately 10–30% of patients go on to develop chronic pain after surgery [8–10]. Given that over 300 million major surgeries are performed globally each year [11], chronic post-surgical pain (CPSP) [10] is associated with a massive economic and societal burden [12]. Perhaps most significant is the impact chronic pain has on the quality of life of individuals who experience it. Patients have reported that beyond physical function, chronic pain interferes with their relationships, social life, sleep and mood [13].

Several factor are associated with an increased risk of developing CPSP [9, 12]. Among these, having pain itself, including chronic pain prior to surgery and intense acute post-surgical pain [8, 14, 15], are among the most commonly-identified risk factors [9, 12, 16]. Higher consumption of analgesics [17, 18], preoperative opioid use [19, 20], as well as psychological factors including distress about bodily sensations [21], depression, anxiety, and pain catastrophizing [22–24] are associated with a higher risk for CPSP. Timely intervention during the perioperative period can address many of these risk factors and help in preventing the transition from acute to chronic pain after surgery, thereby benefitting patients and reducing health care system costs [4, 5, 12]. However, there is a gap in effective care when it comes to preventing and managing pain during the perioperative period. To address this gap, we established the Transitional Pain Service (TPS) at the Toronto General Hospital in Toronto, Canada, in 2014 [4].

### Overview of the TPS model

The TPS model was conceived as a means to intervene during the transitional period, when acute pain after surgery can become chronic, in order to minimize the number of post-surgical patients who develop CPSP and to reduce the use of and dependence on opioids post-surgery [4, 12, 25]. Pain care at a TPS clinic is delivered according to a coordinated multidisciplinary approach that involves medical management, psychological support, and physical rehabilitation. The key aims according to the TPS model are to:

1. Manage pain comprehensively pre, peri, and postoperatively for patients who are at a high risk of developing CPSP.
2. Manage opioid medication and weaning for medically complex patients after post-surgical discharge.
3. Use a multidisciplinary approach to facilitate patient function and improve their quality of life after surgery.

The TPS model places emphasis on the psychological needs of patients [4, 12, 25]. The integration of evidence-based psychological treatment into post-surgical pain management empowers patients with skills that help them regain their daily functioning and quality of life, even if their pain does not resolve completely [26]. The psychology team works with patients to develop personalized pain management plans and to address distress related to pain and associated mental health concerns that may amplify pain. Patients learn behavioral skills to support pain management and opioid weaning and to reduce pain-related disability.

### The TPS at Toronto General Hospital

The Toronto General Hospital is Canada’s leading surgical centre [27], seeing over 6000 patients on a yearly basis, with a particular emphasis on procedures that are known to carry a high risk for CPSP [4]. Currently, the clinical team at the TPS clinic at this hospital consists of anesthesiologists and nurse practitioners with acute or chronic pain-specific training, clinical psychologists, a physical therapist with acupuncture training, a psychiatrist, and a patient care coordinator. Since its founding year, the clinic has seen over 2600 patients, with an average of nearly 300 patients per year over the last five years.

Patients may be referred to the TPS at different time points during the peri-operative period. Patients with complex needs are typically seen before surgery to develop a plan for their peri-operative pain management. As part of these patients’ management plans, physicians may reduce opioid medication pre-operatively by introducing other modes of pain control to improve CPSP outcomes in the post-surgical period [28]. Patients may also be identified with a “pain alert” priority if they present to the pre-surgical anesthesia clinic while taking opioids for a chronic pain condition. These patients are then followed by the TPS after their surgical procedure.

Patients who are not identified as high risk during the pre-admission visit may be referred to the clinic by the Acute Pain Service (APS) post-operatively, according to established referral guidelines [4, 12]. After surgery and prior to post-surgical discharge, TPS nurse practitioners work with patients to treat and optimize in-patient pain care with multimodal analgesia and patient education about pain management. A physician may also assess the patient and propose the opportunity to participate in a multidisciplinary pain care program, which may include medications, physiotherapy, and psychological counselling. For some patients, medical and psychological (e.g., clinical hypnosis [29, 30]) treatment may begin in the hospital prior to discharge.

Most patients identified as needing TPS involvement are followed up by the out-patient clinic, also located at Toronto General Hospital, typically 2–4 weeks after discharge. During the initial visits, the management plan is (re-) assessed and the TPS team works to address each patient’s needs through a multidisciplinary approach to pain care. Patients may be referred for physiotherapy to help them with pain relief and physical rehabilitation to restore function. The pain management plan may also include weaning from opioids, if needed. Patients who require high-dose opioids, who have a history of chronic pain or mental health concerns, or who report a high level of distress and pain may be referred to see a TPS clinical psychologist.

The TPS psychology treatment approach is based primarily in Acceptance and Commitment Therapy (ACT) that is adapted for post-surgical patients and addresses pain coping, pain interference, as well as mood and anxiety issues [31]. ACT is a Third Wave behavioral therapy, having developed out of cognitive behavioral therapy, that incorporates mindfulness and acceptance and emphasizes that personal values shape behavioral choices [32–34]. There is substantial literature showing that ACT improves outcomes for chronic pain patients [32]. The psychology team offers ACT in one-on-one sessions, typically via a 4–6 session protocol. The team has also adapted ACT to be delivered in a one-session group workshop that focuses on both behavioral intervention and pain education.

A recent TPS initiative involves the integration of the “Manage My Pain” (MMP) app as a means to engage patients more directly with self-evaluation and self-management and to enhance patient-clinician communication. This user-driven app was developed and tested in collaboration with the TPS team [35–38]. Patients can use the app to track their pain, symptoms, medications, and daily functioning. The tracked data can be easily shared with clinicians during or between appointments. The app is also used to administer clinic intake and follow-up questionnaires.

Patients followed by the out-patient TPS clinic are typically assessed every 2–4 weeks within the first 3 months of surgery. Physicians adjust analgesic medications including opioids until patients are weaned from opioid medications or until the dose is at a safe level and pain is well controlled. The TPS aims to see patients for 3–6 visits, typically within 6 weeks to 6 months after hospital discharge, before handing over care to their family physicians if possible.

### The impact of the TPS

Since the launch of the clinic, the TPS team has been investigating the impact of the program on patient outcomes. In a preliminary study, TPS patients reported a greater decrease in pain intensity over time and faster trajectory towards mild pain compared to control patients who were not followed by the TPS [39]. In a retrospective study of patients who were given opioids after surgery and followed by the TPS, 46% of the opioid-naive patients and 25% of opioid-experienced patients were completely weaned from opioid medications 6 months after surgery [12, 40] with a concomitant reduction in pain intensity and pain interference scores [12]. The study also showed that the TPS effectively identified patients who were at a high-risk of developing post-surgical pain problems.

The TPS has also demonstrated success in helping complex patients with chronic pain, long-term opioid use, and related comorbidities. Case studies involving these patients show the effectiveness of the multidisciplinary approach at reducing opioid use and pain interference, as well as supporting recovery of function and improving quality of life [41–43].

Preliminary evaluation of the TPS clinical psychology program showed that TPS patients who received ACT had a greater reduction in pain interference, depressed mood, and opioid use compared to those who did not undergo ACT. Both groups showed an overall reduction in pain, pain interference, pain catastrophizing, anxiety and opioid use by their last TPS visit [12, 31]. A recent study by the TPS psychology team also demonstrated that clinical hypnosis had an opioid sparing effect during the acute postoperative period and protected against increases in pain catastrophizing at one-week after surgery [29]. Furthermore, in a study of the effectiveness of the MMP app, individuals who used the app reported lower anxiety and greater reduction in pain catastrophizing compared to a group of patients who did not use the app [36].

The evaluation of the impact of the TPS is ongoing. The TPS team at Toronto General Hospital is currently conducting a multi-site randomized controlled trial to evaluate the efficacy of the TPS model at a larger scale, across five different sites in Ontario.

## Knowledge translation initiatives at the Transitional Pain Service

**Fig. 1:**
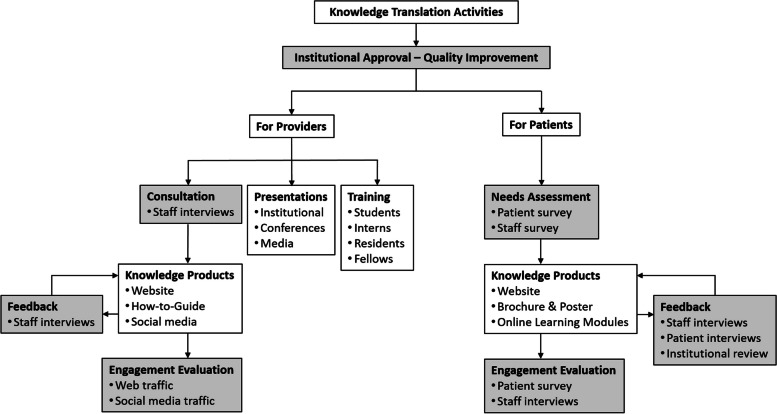
Overview of knowledge translation activities at the Transitional Pain Service (TPS) for healthcare providers and patients. Boxes in gray indicate consultation, feedback, and evaluation activities

One of the aims of the TPS as a pioneering clinic in transitional pain care is to increase awareness and acceptance of multidisciplinary pain care during the peri-operative period among both healthcare providers and patients. To accomplish this aim, we have conducted knowledge translation activities geared towards these two groups, summarized in Fig. 1. These activities were supported by a major grant from Health Canada’s Substance Use and Addictions Program to evaluate and promote this model of care.

### Knowledge translation approach

Our knowledge translation activities follow the Knowledge to Action (KTA) framework [44, 45]. This framework is widely used in healthcare settings and recommended for use in the field of chronic pain [46]. It describes how information (knowledge) can be used to affect change (action) and is thus a potent tool for developing impactful knowledge products and educational programs for both healthcare practitioners and patients [45].

The KTA framework consists of 2 components: a rotating knowledge creation funnel, and an action cycle. The knowledge creation funnel consists of three overlapping phases: 1) knowledge inquiry, 2) knowledge synthesis, and 3) knowledge products and tools. Concurrent with, or subsequent to, the knowledge creation process is the action cycle, which consists of 7 phases. Importantly, the phases do not need to be completed in order, and several may be completed simultaneously or in an overlapping fashion. Graham et al. [45] emphasize the cyclical nature of the process, which implies a constant effort towards improvement. The cycle involves: 1) determining the area of change, selecting the specific knowledge relevant to the identified area, and determining the gap between the knowledge and actual practices, 2) adapting knowledge given cultural, social, and economic contexts, 3) assessing the barriers and facilitators to implementation of the knowledge, 4) selecting implementation strategies appropriate to the setting, 5) monitoring how the knowledge is being used an implemented, 6) evaluating outcomes, 7) sustaining changes and knowledge implementation.

A hallmark of the KTA framework is the proper consideration of all stakeholders and understanding the social context in which the knowledge will be implemented. Ultimately, the processes described in the framework are fluid and can be used as a roadmap for collective action by any group [45, 46].

### Applying the KTA framework at the TPS

The development of the TPS model and establishment of the Toronto General Hospital TPS clinic is a prime example of knowledge (i.e., evidence for effectiveness of a multidisciplinary approach in pain care) being translated into action and implemented within the existing healthcare structures. In keeping with the cyclical nature of the process of knowledge translation, the TPS clinic not only engages in ongoing evaluation of its impact but also in further knowledge translation to promote acceptance of the model by key stakeholders (i.e., healthcare practitioners and patients) by developing and disseminating targeted resources and tools. The following sections outline the steps taken with each stakeholder group, including determining the knowledge gap, adapting the knowledge to the local context, and monitoring knowledge use. We provide details of these knowledge translation activities together with their evaluation outcomes, where relevant.

### Institutional approval

The knowledge translation initiatives conducted at the TPS clinic are part of an institutionally approved Quality Improvement project. All activities described here that involved input from TPS clinic staff and patients (i.e., surveys, interviews) were approved by the Quality Improvement Review Committee (QIRC) at the University Health Network (UHN), including protocols and informational consent (QI ID#: 21–0310). QIRC determined that the project falls outside the scope of research requiring Research Ethics Board (REB) review, as described in the Canadian Tri‐Council Policy Statement V.2. This determination was subsequently reviewed and confirmed by a UHN REB Chair.

The need for signed consent to participate was deemed unnecessary as part of the approval by QIRC (QI ID#: 21–0310) and in accordance with Canadian Tri‐Council Policy Statement V.2. An informational consent letter was provided instead to all participants, which outlined the objective of the project, privacy and confidentiality, and contact information for the project team and QIRC.

### Initiatives geared towards healthcare providers

Implementation of a multidisciplinary pain care model fundamentally relies on awareness and acceptance of the model by healthcare providers delivering pain care across the health system, from pain specialists to primary care physicians, nurses, and allied healthcare providers. However, gaps in knowledge and training are known barriers to adopting this model broadly [3]. Supporting the training and education of healthcare providers is a key priority of the knowledge translation initiatives at the TPS.

#### Education and training of healthcare providers

The Toronto General Hospital TPS program does not function as a stand-alone clinic, but instead is integrated within existing systems and practices at the hospital, including pre-admission, surgical care, and acute pain management. To effectively integrate the program into the existing system, knowledge translation initiatives at the local level were essential. Members of the TPS clinical team conducted seminars, rounds presentations, ward in-services, and informal discussions aimed towards clinicians in other departments to introduce and explain the TPS program and its goals.

Furthermore, our program regularly trains national and international clinical fellows in medicine and psychology who are looking to specialize in multidisciplinary and transitional pain care. The clinic also regularly hosts medical trainees including medical students and residents. Our Psychology Service offers internships and other training opportunities to clinical psychology trainees. Since the establishment of the clinic, we have trained 16 medical fellows in anesthesia and pain medicine, 3 nurse practitioners, and 10 clinical psychologists.

#### Resources and knowledge tools for healthcare providers

We have engaged in extensive outreach to healthcare providers outside of our hospital network through a number of knowledge translation activities. Our team regularly participates in lectures, seminars, and workshops at the local, national, and international level as well as opportunities to discuss and promote our care model in the local and national media. We also use our social media account on X (formerly Twitter) [47] to communicate with the broader healthcare community.

We have also developed a website for the program that provides information geared specifically towards healthcare providers [48]. The content of the website was developed following consultation with members of our clinic staff representing different professions, including physicians, psychologists, physiotherapist, and administrative staff. Clinic staff also provided feedback throughout the development process. The completed website provides general information about our program, guidelines regarding risk factors for CPSP, and referral information. Furthermore, the website provides a link to our “How-To-Guide to Establish a TPS” [49]. This document serves as a step-by-step tool for providers interested in developing their own multidisciplinary program within their healthcare setting. All the information featured in our online resources is available in English and French.

#### Impact of KT activities for healthcare providers

We have had substantial engagement with our online resources geared towards healthcare providers. Our web pages for health professionals have had more than 3,500 views out of a total of over 38,000 views for the entire website since its launch in July 2021. Our how-to-guide for establishing a TPS has been downloaded over 330 times.

A key indicator of the success of the TPS model is the establishment of numerous new TPS clinics. In Canada, there are now TPS programs in Toronto, Vancouver, BC, Calgary, AB, Montreal, QC, and soon to be established clinics in Thunder Bay, ON and St. Johns, NL. Internationally, many programs have now been established in the U.S.A in both academic medical centres and Veteran’s Affairs Hospitals (e.g., Vanderbilt, Utah, Duke, and Emory Universities) as well as globally (e.g., Australia, Germany, Mexico, Netherlands, Norway, and the UK). We have created a living map of existing TPS clinics on our website [50]. Visitors to the site can add their program info to be included on the map.

### Initiatives geared towards patients

Patients often hold misconceptions about chronic pain, which can interfere with their engagement with interventions and active participation in treatments [51]. An important component of multidisciplinary pain care is providing education to patients about chronic pain as a biopsychosocial phenomenon and the benefits of the multidisciplinary approach to chronic pain management [51, 52], including around the peri-operative period [53].

Prior to developing patient education resources at the TPS, we conducted a needs assessment to provide us with greater insights into our patients’ understanding of pain, expectations for pain management, and knowledge of TPS services offered. The patient needs assessment included one staff and one patient survey.

#### Patient needs assessment – staff survey

We sent an online Google Forms survey to 15 patient-facing staff at the Toronto General Hospital TPS clinic. The survey was developed internally and included 9 multiple choice or multi-select questions and fields to provide additional comments. The questions covered three main topics: perspectives on patient education at the TPS, overcoming barriers to successful pain management through patient education, and areas of focus for patient education. Eleven staff provided responses to the survey, including physicians, nurse practitioners, psychologists, and clinic administrative staff. Table 1 provides an overview of survey questions and responses. As this is a small internal sample of staff, their survey responses provide insights relevant to one clinic and may not directly generalize to other clinical contexts.

**Table 1:** Staff responses to needs assessment survey

Survey Questions	Available Response Options	(n) Endorsed*
How much patient education are TPS patients currently getting?	None	0
	A Little	6
	Some	2
	A Lot	0
	I'm not sure	3
Do you engage in any patient education (formal or informal) as part of your interactions with TPS patients?	Yes	7
	No	2
	Not applicable	2
If you do provide educational resources to TPS patients, do they engage well with these resources?	Yes	4
	No	0
	I'm not sure	4
	Not applicable	3
Is there a systematic approach to patient education at the TPS?	Yes	1
	No	5
	I'm not sure	5
What are common barriers to successful pain management that can be addressed through patient education at the TPS?	Poor understanding of how pain works in the body	8
	Poor understanding of the biopsychosocial perspective on pain	10
	Poor understanding of the pain management triad (medicine, movement, mind)	10
	Focus on medication as the only effective pain management strategy	8
	Belief that health practitioners are the only ones who can manage pain (i.e., patients are passive recipients)	10
	Free form: Limited expectation management from surgical team	1
	Free form: Passivity—not asking for options other than medication	1
	Free form: Past history of addiction or complex psychosocial history	1
Are there any specific types of treatments or pain management approaches that TPS patients are often hesitant to participate in?	Psychology	7
	Physiotherapy	2
	Acupuncture	3
	Tracking symptoms (e.g., Manage My Pain App)	5
	Active engagement in pain management strategies	9
	Interventional procedures	2
What topics would TPS patients benefit from knowing more about?	How pain works in the body	8
	The role of the brain in pain	10
	How medications work for managing pain	6
	How pain blocks work for managing pain	5
	The effectiveness of psychological treatment for pain management	10
	The importance of movement and activity for pain management	11
	Importance of self-driven pain management	7
	The role of the situational context in pain	5
	The importance of mindset for pain management	7
	How mindfulness helps in pain management	7
	Free form: Barriers to managing pain	1
What approaches would improve TPS patient engagement with educational resources?	Clinician-led education—Individual	5
	Clinician-led education—Group workshops	9
	Peer-led education—Individual	1
	Peer-led education—Group workshops	4
	Self-directed education (e.g., online module)	10
Which of the following media do you think would be most suitable to provide educational resources to TPS patients?	Paper brochures	5
	Written information on a website	3
	Video on a website	9
	Interactive module on a website	10
	Podcasts	6
	Free form: All of the above	1

^*^Total of 11 responders

Most clinic staff reported that they engage in patient education as part of their interaction with patients, but that patients are only getting “some” or “little” pain education overall. Staff were mixed on whether patients engage well with the educational resources provided. Finally, only one staff respondent indicated that the TPS has a systematic approach to patient education, whereas all others indicated that it does not or that they were not sure.

When asked about barriers to successful pain management that can be addressed through pain medication, most clinic staff selected “poor understanding of the biopsychosocial perspective on pain”, “poor understanding of the pain management triad (medicine, movement, mind)”, and “belief that health practitioners are the only ones who can manage pain”. When asked about pain management approaches that patients are hesitant about, the most common responses pointed to psychology treatments and active engagement in pain management. Similarly, when asked about topics of pain education that patients would most benefit from, most staff selected “the role of the brain in pain”, “the effectiveness of psychological treatment”, and “the importance of movement and activity”. In free form responses, staff reiterated that patient education should focus on non-pharmacological and non-interventional approaches to self-management of pain.

With regards to the format of patient education, clinic staff selected clinician-led group workshops and self-directed education through online learning modules as the best approaches to improve patient engagement with educational resources. Videos and interactive modules on a website were selected as the most suitable media to deliver patient education.

#### Patient needs assessment – patient survey

Patients registered with the Toronto General Hospital TPS clinic between Jan 2021 and July 2022 were invited to respond to the patient needs assessment survey. The survey was administered via the MMP app or email using the REDCap electronic data capture platform [54, 55] hosted at University Health Network, as well as in paper format to patients who did not provide electronic contact information. The survey was internally developed and included 18 questions regarding patient views about pain, outlook on managing pain, understanding of the multidisciplinary pain treatment approach at the TPS, and preferences for accessing educational resources. Patients were not required to answer every question. 15 questions requested patients to respond to a statement on a 5-point scale, from 1 (“strongly agree”) to 5 (“strongly disagree”). Remaining questions were presented in a multiple choice or multi-select format. Of 252 patients contacted via the MMP app, 57 responded to the survey. Of 84 contacted via email, 26 responded. Additionally, 6 patients completed the survey on paper. The overall response rate was 26%. As this is a relatively small internal sample of patients, their survey responses provide insights relevant to one clinic and may not directly generalize to other clinical contexts.

Table 2 provides an overview of survey questions and responses for patients’ views about pain and pain management. The responses demonstrate that patients’ views are somewhat congruent with the current knowledge about pain and evidence-based approaches to pain management. Most patients (> 90%) agreed that: all pain is real, it’s important to learn how pain works in the body, mindset affects coping with pain, and patients should have an active role in pain management. There was less agreement among patients (70–90%) that: pain is a protective feeling, pain is not always related to tissue damage, how pain feels can depend on the situation, and movement is helpful in managing pain. Patients were also in agreement with the statement that “pain is a sign that something is wrong in the body”. This statement is incongruent with the current understanding of pain signaling, especially when it comes to chronic pain, though the phrasing of the statement may not have provided enough context for this interpretation. There was also a mixed agreement about the statement that “the most effective way to manage pain is by taking pain medication”. These responses demonstrate that patients at the TPS are relatively knowledgeable when it comes to understanding pain and pain management, but that pain education in some areas is still warranted.

**Table 2:** Patient responses to needs assessment survey

Survey Questions	Available Response Options	Mean*	SD	% Agree ~
What are your views about pain?	Pain is a sign that something is wrong in the body (reverse coded)	1.7	0.8	89
	Pain is a protective feeling in the body	2.1	0.9	72
	All pain is real no matter what is causing it	1.8	0.7	91
	Pain is not always related to tissue damage in the body	2.0	0.9	82
	How much pain you feel depends on the situation you are in at the time	2.2	1.0	75
What are your views about managing and coping with pain?	The most effective way to manage pain is by taking pain medication (reverse coded)	2.8	1.1	44
	It's important to learn how pain works in the body to cope with pain	1.8	0.7	91
	Movement and physical activity are helpful in managing pain	2.1	1.0	71
	Your mindset towards pain affects how you cope with pain	1.8	0.8	90
	Patients should have an active role in their pain management	1.4	0.6	98
What are your expectation about managing your pain?	I feel confident in managing my pain	2.4	1.0	61
	I expect that by the end of my treatment at the TPS my pain will go away completely	3.4	1.1	24
	I expect that by the end of my treatment at the TPS I will have the skills needed to manage my pain	2.4	1.0	60
About your experiences with Psychology services	I have a good idea about how psychological coping strategies fit into my overall pain management plan	2.6	1.1	67
	I feel confident in using psychological coping strategies at home	2.4	1.1	80

^*^Responses on a 5-point agreement scale; 1 = Strongly agree, 5 = Strongly Disagree

~ Refers to % of patients who responded Strongly agree & Agree

In terms of expectations about managing pain, 61% of patients agreed that they felt confident in managing their pain and 60% agreed that they will have the skills needed to manage their pain by the end of their treatment at the TPS. Few patients (24%) agreed that their pain will go away completely by the end of their treatment.

When asked about familiarity with the multidisciplinary services available at the Toronto General Hospital TPS, only 34% of patients heard about psychology services, 36% heard about physiotherapy services, and 20% heard about acupuncture being available as a complement to physiotherapy. Furthermore, only 18% of patients reported having seen a psychologist at the TPS or another clinic. When these patients were asked about their experiences with TPS psychology services, 80% agreed that they felt confident in using psychological coping strategies, but only 67% agreed that they have a good idea about how psychological coping strategies fit into their pain management plan. These responses demonstrate that there is little familiarity with the multidisciplinary services offered at the TPS and that patients who have seen a psychologist may benefit from having more information about how psychology fits into the treatment approach at the clinic.

When asked about what media patients would prefer to use to learn more about pain management, 58% chose a video on a website, 57% chose written information on a website, 42% chose an interactive application on a website, 28% chose paper brochures, 23% chose podcasts, and 3% chose other media.

Overall, the patient needs assessment findings showed that patients at the Toronto General Hospital TPS clinic recognized the importance of learning about pain and taking an active role in pain management. Although most patients understood the current perspectives on pain and pain treatment approaches, many still saw medication as the most effective way to manage pain. Only over half of the patients surveyed felt confident about managing their pain and learning to do so at the TPS. Many patients who completed the needs assessment had not heard about the services offered at the TPS and only a small percentage had seen a pain psychologist. Most patients preferred educational resources offered through an online medium.

#### Educational resources for patients

The needs assessment demonstrated that there was a need to provide more resources to patients to support their treatments at the clinic, particularly information regarding the multidisciplinary approach to pain management and the role of psychology in pain care. In response to these findings, the TPS team set out to develop a set of educational resources for patients. As both patients and staff indicated a preference for resources available online, efforts were focused on this medium as the primary access point for resources.

A dedicated page for patients and families [56] was developed on our TPS website to direct visitors to separate pages that provided information about the different services available at the TPS clinic, including information about the multidisciplinary approach to pain care, psychology and physiotherapy services, digital tools, and learning resources. In partnership with the Toronto Academic Pain Medicine Institute (TAPMI), the main referral hub for pain clinics in the city of Toronto, the TPS team also developed a web page on the TAPMI website [57]. This web page houses introductory information about CPSP for patients and directs them to the main TPS website.

Two in-depth TPS online learning modules were developed, titled “Learn about Ongoing Pain after Surgery” and “The Role of Psychology in the Treatment of Pain”. These modules were created using Articulate Rise 360 (www.articulate.com), an authoring software for online learning tools. This software was selected for its user friendly and accessible interface for learners and many interactive features. Module development incorporated feedback on the content and navigation from TPS staff, four TPS patients, two patient volunteers with chronic pain at Toronto General Hospital not followed by the TPS, as well as staff at the University Health Network Patient Education and Engagement office. The learning modules are housed on the learning resources page of the TPS clinic website.

The final version of each module is composed of text, video, and audio content to support multi-modal learning. The first learning module contains information regarding the typical progression of pain after surgery followed by an overview of how pain works in the body and how it may become chronic after surgery. Risk factors for CPSP are also discussed and information is provided about multidisciplinary management of CPSP. Finally, a list of further resources and tools is provided at the end. The second learning module provides an overview of the pain treatment approach at the TPS followed by an introduction to how psychology treatment can help with pain. Next, psychology treatment strategies used at the TPS are presented as well as a typical timeline for treatment. Finally, a list of resources and companion tools is included. The modules contain several learning checks in the form of brief quizzes to help support retention of the presented material.

To direct patients to online resources, we also developed paper resources to disseminate at the clinic, including a brochure and posters. These resources contain introductory information as well as a link and QR code that directs patients to the learning resources on the TPS website. Clinicians are encouraged to point patients to these resources during regular appointments. We have also added a link to our resources to the appointment reminder emails that patients receive from the clinic.

#### Patient engagement with educational resources

To monitor knowledge use, we used web analytics tools to track access to the knowledge products available online. The web pages for patients and families on the TPS website have received over 5,000 views since the launch of the website in July 2021. The online learning resources page has been viewed nearly 800 times and the learning modules have been accessed over 120 times.

In addition to web analytics, we also conducted a survey among TPS patients to gauge their engagement with the available online resources. Pre- and post-surgical patients referred to the TPS in 2022 and 2023 who had at least two visits with a TPS physician were invited to respond to an online patient engagement survey administered using the REDCap platform [54, 55]. The survey asked patients if they visited the TPS website and online learning modules (e.g., “Have you visited the Transitional Pain Service website?”) and if not, the reasons they had not visited (e.g., “I did not know about the website”, “I do not know how to use the website”, “I am not interested in the website’, etc.). Of 146 patients invited to participate, a total of 47 fully or partially completed the survey (32%). Twenty-two out of 47 patients (47%) reported that they visited the Transitional Pain Service website, 20 out of 45 patients (44%) accessed the “Learn About Ongoing Pain After Surgery” module, while 11 out of 43 patients (26%) accessed the “The Role of Psychology in the Treatment of Pain” module. The most common reason for not visiting the website or viewing the learning modules was that patients did not know about them. Additionally, several patients reported that they were not interested in using the online resources or did not know how to use them.

To further understand facilitators and barriers to patient engagement with the educational resources available at the clinic, we conducted interviews with eight of our TPS clinic staff, including physicians, nurse practitioners, psychologists, and administrators. The list of interview questions can be found in Supplementary Material S1. Using a thematic analysis approach, we identified three main barriers to patient engagement that emerged from staff responses to the interview questions. The first barrier concerned staff having limited time during appointments to discuss the educational resources with patients. Staff discussed the importance of introducing patients to the educational resources during clinic appointments but acknowledged that time constraints often leave insufficient opportunity to do so. The second barrier was the lack of familiarity with the content of the resources. This barrier prevented some staff from promoting the resources to patients or discussing how patients could benefit from them. The third barrier related to concerns regarding accessibility of the resources. Staff noted that some patients have limited access to the internet while others, especially older patients, are apprehensive about using digital resources. They also emphasized the importance of using accessible language in the resources and ensuring readability.

Overall, our team continues to monitor the use of learning resources by our patients and is implementing strategies to enhance awareness and access to these resources. These strategies include developing resources in paper format and providing printed note cards to remind clinicians to recommend the resources to patients.

## Discussion

The knowledge translation activities at the TPS at Toronto General Hospital have been at the core of promoting the program and enhancing its acceptance among healthcare providers and patients. The KTA framework has been an essential tool in providing a roadmap for organizing and coordinating the various knowledge translation initiatives in a systematic manner, from assessing needs, developing, adapting, and implementing knowledge products to monitoring knowledge use. Through these activities, the TPS team has also been able to engage more effectively with stakeholders and continue to build on this engagement to ensure the sustainability of the TPS model.

Our knowledge translation initiatives have been well-received by healthcare providers. Outreach to this group has focused on speaking engagements by our team at seminars and conferences, as well as through our online presence. A testament to the success of our efforts is the establishment of various TPS clinics at other institutions across Canada, the USA, and globally. However, challenges remain in supporting the growth of this model as part of standard perioperative pain care. There is a need to train more clinicians specializing in transitional pain and CPSP and provide more educational resources to primary physicians. To this end, the American Society of Regional Anesthesia and Pain Medicine (ASRA) created a special interest group in order to dedicate specific attention towards the topic of chronic post-surgical pain [58]. In Canada, our team partnered with Pain BC/Pain Canada to contribute to developing educational modules geared towards healthcare providers that focus on pain and surgery [59]. As part of the same partnership, we also contributed to developing an online module for patients undergoing surgery that focuses on pain management after surgery, risks of having CPSP, and strategies to mitigate pain-related disability [60]. In addition, we are developing tools to support practitioners in implementing psychology interventions. Our team has partnered with the developers of the MMP app to create a self-guided ACT intervention based on our psychologist-guided group workshop. The aim is to provide this tool as a way to implement the ACT intervention at clinics that have limited capacity to offer psychologist-led services.

Engaging patients with our knowledge translation initiatives has required sustained outreach at our clinic. We initially focused on developing resources in an online format. However, continued tracking of engagement revealed that patients were not readily accessing them. We then supplemented our online content with printed brochures and posters at the clinic to direct patients to the online resources. We also encouraged our clinic staff to ensure that they discuss the available resources with patients. Feedback from staff indicated that limited time during appointments is often a barrier to providing patient education and discussing how to access our online resources. In response to this feedback, we are creating paper postcards with basic information and links that can be easily distributed to patients during an appointment. We are also working to provide our online content in paper format to offer more options for access to patients.

In addition to our initiatives geared towards providers and patients, we are also working to engage policy makers to draw greater attention to the TPS approach. We have received funding from Health Canada, the national governmental health body in Canada, which has supported our knowledge translation initiatives. We also recently held a TPS Knowledge Exchange conference and presented at the National Pain Congress organized by Health Canada to engage directly with policy makers at different levels of government and to highlight our achievements over the past 10 years. Our goal is to raise awareness among policy makers about the benefits of the TPS model in order to ensure a sustained investment in the future development and growth of this approach to perioperative pain care.

### Insights and recommendations

The following are the key insights and recommendations for teams undertaking knowledge translation initiatives based on our experiences at the TPS clinic at Toronto General Hospital:

1. Follow a framework for knowledge translation activities. We recommend that teams adopt a framework (e.g., KTA framework) early on in their initiative to support a systematic approach to developing and evaluating knowledge translation activities.
2. Prioritize accessibility of resources. We recommend that teams consider the accessibility needs of their target population at the onset of an initiative and plan for different formats of knowledge products (e.g., web, interactive digital, paper, short & long versions) and multimodality of resources (e.g., text, video, audio) when possible.
3. Implement various assessment and evaluation methods. We recommend that teams consider both qualitative (e.g., interviews, focus groups) and quantitative (e.g., surveys, web usage metrics) assessment methods in evaluating initiatives and knowledge products to obtain more nuanced and actionable feedback.
4. Cultivate connections and partnerships for effective knowledge dissemination. We recommend that teams cast a wide net when considering dissemination strategies and engage with various partners, associations, and stakeholders to enhance the impact of knowledge translation initiatives.

### Limitations

The findings presented here are limited to one transitional pain clinic, the limited sample of patients and healthcare providers at the clinic who participated in the different phases of this project, and the data collection approaches that were feasible given the resources available at the clinic. Therefore, the findings and recommendations may not directly generalize to other clinical contexts. Each medical clinic is situated in a unique institutional environment and healthcare system that influences the types of knowledge translation initiatives that are feasible and practical. However, following the KTA framework, knowledge translations activities can be adapted to each unique context and their impact evaluated along the way to make adjustments as needed.

## Conclusion

Implementation of a multidisciplinary chronic pain care model relies on awareness and acceptance of the model by healthcare providers and patients. Our ongoing knowledge translation activities at the Toronto General Hospital TPS have contributed to promoting this model of care in the peri-operative period and we hope that the findings presented here will serve as a useful guide for other clinical teams interested in undertaking similar activities. The key insights from our initiatives highlight the importance of having a systematic framework for knowledge translation, prioritizing accessibility of resources, implementing various evaluation methods, and leveraging professional networks for resource dissemination. Continued investment in education and sharing knowledge is key to sustaining the growth of the multidisciplinary approach to chronic pain care.

## Supplementary Information

Supplementary Material 1.

## Data Availability

The datasets generated and analysed during the current study are available from the corresponding author on reasonable request.
