# Supplementary material for: Knowledge translation initiatives at the Transitional Pain Service: insights from healthcare provider outreach and patient education
Source: BMC Health Serv Res. 2025 Jan 29;25:169. doi: 10.1186/s12913-025-12301-y (PMC11776314; doi:10.1186/s12913-025-12301-y)
Supplement: Supplementary file 1 — Supplementary Material 1. [file 12913_2025_12301_MOESM1_ESM.docx]

**Supplementary Material S1**

**Interview Questions for Transitional Pain Service (TPS) staff**

1. Can you describe the process for when a patient is first introduced into the clinic for treatment?
2. What steps are involved in the patient registration process?
   1. Are TPS resources consistently being introduced at this stage? If so, how?
3. What methods are currently used to educate patients about their condition and treatment options?
   1. Are TPS resources introduced to patients during this stage? If so, how are they being introduced, and how many patients are being introduced?
4. Are there any challenges in having you recommend TPS resources available to patients, such as educational materials, apps, or support groups?
5. Are TPS resources discussed with patients as part of the discharge plan, and if so, how?
6. Are there mechanisms in place to encourage patients to continue using TPS resources after leaving the clinic?
7. What ideas do you have for further enhancing patient engagement with TPS resources in the future?
